# *Materials* Best Paper Award 2014

**DOI:** 10.3390/ma7021441

**Published:** 2014-02-24

**Authors:** Maryam Tabrizian

**Affiliations:** Department of Biomedical Engineering, Faculty of Medicine/Faculty of Dentistry, Duff Medical Science Building, McGill University, Room 313, 3775 University Street, Montreal, QC, H3A 2B4, Canada; E-Mail: maryam.tabrizian@mcgill.ca

*Materials* instituted an annual award in order to acknowledge outstanding papers in the area of materials science and engineering published in *Materials*.

Nominations were selected by the Section Editor-in-Chiefs of *Materials* from all papers published in 2010. The awards are issued to reviews and articles respectively. We are pleased to announce the second “*Materials* Best Paper Award” for 2014 for which the following five papers were chosen:

**Article Award:**

*1^st^* *Prize*

**Andreas Klein, Christoph Körber, André Wachau, Frank Säuberlich, Yvonne Gassenbauer, Steven P. Harvey, Diana E. Proffit and Thomas O. Mason**

Transparent Conducting Oxides for Photovoltaics: Manipulation of Fermi Level, Work Function and Energy Band Alignment

*Materials* **2010**, *3*(11), 4892-4914; doi:10.3390/ma3114892

Available online: http://www.mdpi.com/1996-1944/3/11/4892

*2^nd^* *Prize*

**Aneta Slodczyk and Philippe Colomban**

Probing the Nanodomain Origin and Phase Transition Mechanisms in (Un)Poled PMN-PT Single Crystals and Textured Ceramics

*Materials* **2010**, *3*(12), 5007-5028; doi:10.3390/ma3125007

Available online: http://www.mdpi.com/1996-1944/3/12/5007

*3^rd^* *Prize*

**Osama Shekhah**

Layer-by-Layer Method for the Synthesis and Growth of Surface Mounted Metal-Organic Frameworks (SURMOFs)

*Materials* **2010**, *3*(2), 1302-1315; doi:10.3390/ma3021302

Available online: http://www.mdpi.com/1996-1944/3/2/1302

**Review Award:**

*1^st^* *Prize*

**Lutz-Christian Gerhardt and Aldo R. Boccaccini**

Bioactive Glass and Glass-Ceramic Scaffolds for Bone Tissue Engineering

*Materials* **2010**, *3*(7), 3867-3910; doi:10.3390/ma3073867

Available online: http://www.mdpi.com/1996-1944/3/7/3867

*2^nd^* *Prize*

**Koen Van den Eeckhout, Philippe F. Smet and Dirk Poelman**

Persistent Luminescence in Eu^2+^-Doped Compounds: A Review

*Materials* **2010**, *3*(4), 2536-2566; doi:10.3390/ma3042536

Available online: http://www.mdpi.com/1996-1944/3/4/2536

We believe that these five exceptional papers are valuable contributions to *Materials* and the scientific research area. On behalf of the Prize Awarding Committee and the Editorial Board of *Materials*, we would like to congratulate these five teams for their excellent work. In recognition of their accomplishment, for the article group, Dr. Andreas Klein, Dr. Philippe Colomban and Dr. Osama Shekhah will receive prizes of 600 CHF, 400 CHF and 200 CHF, respectively, and the privilege of publishing an additional paper free of charge in open access format in *Materials*, after the usual peer-review procedure. For the review group, Dr. Aldo R. Boccaccini and Dr. Koen Van den Eeckhout will receive the privilege of publishing a research article free of charge in *Materials* following the peer-review process.

Prize Awarding Committee

*Editor-in-Chief*, Section ‘Biomaterials’

**Prof. Dr. Aldo R. Boccaccini**

Institute of Biomaterials, Department of Materials Science and Engineering, University of Erlangen-Nuremberg, 91058 Erlangen, Germany, E-Mail: aldo.boccaccini@ww.uni-erlangen.de

*Editor-in-Chief*, Section ‘Materials for Energy Applications’

**Prof. Dr. Thomas Lippert**

Materials Group, Research Department General Energy, Paul Scherrer Institut, CH-5232 Villigen, Switzerland, E-Mail: thomas.lippert@psi.ch

*Editor-in-Chief*, Section ‘Structure Analysis and Characterization’

**Prof. Dr. Jung Ho Je**

Department of Materials Science and Engineering, Pohang University of Science and Technology, San 31 Hyojadong, Pohang, 790-784, Korea, E-Mail: jhje@postech.ac.kr

*Editor-in-Chief*, Section ‘Porous Materials’

**Prof. Dr. Rafael Luque**

Departamento de Quimica Organica, Universidad de Cordoba, Campus de Rabanales, Edificio C-3 (Marie Curie-Anexo), Carretera Nacional IV-A, Km. 396, 14014–Cordoba, Spain, E-Mail: q62alsor@uco.es

## Note Added by the Publisher on 1 April 2014



