# Supplementary material for: Materials Best Paper Award 2014
Source: Materials (Basel). 2014 Feb 24;7(2):1441–3. doi: 10.3390/ma7021441 (PMC5453069; doi:10.3390/ma7021441)
Supplement: Supplementary file 1 [file materials-07-01441-s001.docx]

Note Added by the Publisher on 1 April 2014

In regards to the selection process of the Materials Best Paper Awards 2014, the editorial office of Materials and the Prize Awarding Committee would like to clarify the following: Prof. Dr. Aldo R. Boccaccini has not been involved in the selection of the award recipients for the Review Awards category, as he himself appeared as the co-author of one of the candidate review papers for the prize. His contribution was limited to the selection of the Article Awards recipients.
